# Microgroove and Collagen-poly(ε-caprolactone) Nanofiber Mesh Coating Improves the Mechanical Stability and Osseointegration of Titanium Implants

**DOI:** 10.3390/nano7060145

**Published:** 2017-06-13

**Authors:** Morshed Khandaker, Shahram Riahinezhad, Wendy R. Williams, Roman Wolf

**Affiliations:** 1 Department of Engineering & Physics, University of Central Oklahoma, Edmond, OK 73034, USA; sh_riahi@yahoo.com; 2 Department of Pathology, University of Oklahoma Health Sciences Center, Oklahoma City, OK 73034, USA; Wendy-R-Williams@ouhsc.edu; 3 Veterans Affairs Department, Oklahoma City, OK 73105, USA, roman.wolf@va.gov

**Keywords:** titanium, bone, shear strength, in vivo study, electrospun nanofiber

## Abstract

The effect of depositing a collagen (CG)-poly-ε-caprolactone (PCL) nanofiber mesh (NFM) at the microgrooves of titanium (Ti) on the mechanical stability and osseointegration of the implant with bone was investigated using a rabbit model. Three groups of Ti samples were produced: control Ti samples where there were no microgrooves or CG-PCL NFM, groove Ti samples where microgrooves were machined on the circumference of Ti, and groove-NFM Ti samples where CG-PCL NFM was deposited on the machined microgrooves. Each group of Ti samples was implanted in the rabbit femurs for eight weeks. The mechanical stability of the Ti/bone samples were quantified by shear strength from a pullout tension test. Implant osseointegration was evaluated by a histomorphometric analysis of the percentage of bone and connective tissue contact with the implant surface. The bone density around the Ti was measured by micro–computed tomography (μCT) analysis. This study found that the shear strength of groove-NFM Ti/bone samples was significantly higher compared to control and groove Ti/bone samples (*p* < 0.05) and NFM coating influenced the bone density around Ti samples. In vivo histomorphometric analyses show that bone growth into the Ti surface increased by filling the microgrooves with CG-PCL NFM. The study concludes that a microgroove assisted CG-PCL NFM coating may benefit orthopedic implants.

## 1. Introduction

Titanium (Ti) alloy implants are one of the most widely used implants for orthopedic (e.g., hip replacements, plates for the fixation of broken bones) and orthodontic (e.g., implants, braces) surgeries because of its strong mechanical, chemical, and biological properties [1]. An ideal implant for these surgeries should ensure lifelong mechanical stability and induce osseointegration with the adjoining tissues [2]. Ti implant loosening usually occurs from the lack of osseointegration of the implants with the bone. The enhancement of osseointegration of Ti implants with bone is required to improve the implant survival and reduce the number of revision surgeries. Although considerable advances have been made in improving the surface properties of the Ti alloy, the optimum biological performances of Ti alloy implants have not been achieved. The motivation of this study is to improve the titanium implant surface topography, allowing for increased osseointegration of the implants that will provide greater mechanical stability.

Surface geometry and biocompatibility determine the interaction of cells and proteins with the implant surface [3]. Micron to nanometer size grooves can control the cell settlement on implant surfaces or be used to direct tissue generation at the implant/bone interface [4]. A variety of implant surface modifications, including surface cutting, etching, and ion deposition techniques, have been studied and facilitate the formation of an extracellular matrix on the implant surface [4,5,6,7]. Micron to nanosize texturing on the implant is one of the simplest, most scalable, inexpensive, and supplementary surface treatment tools employed to direct the bone response at the interface between an implant and the bone tissue [8,9,10,11,12]. Plasma-surface texturing techniques such as plasma spray and plasma immersion ion deposition have the ability to selectively enhance surface roughness, by approximately several hundred nanometers [13]. Grooves on implants induce a higher amount of osteoblast cell function [14] and implant–bone contact area [15] compared to implants without grooves. Another way to improve the osseointegration of an implant is to coat the implant with a functional biomaterial coating that creates an extracellular matrix on the implant [16]. An extracellular matrix provides the principal means by which mechanical information is communicated between tissue and cellular levels of function [17]. These mechanical signals play a central role in controlling cell fate and establishing tissue structure and function [17]. Implant surfaces can also be improved by mimicking the natural extracellular matrix of bone tissue, which is a highly organized nano-composite [6]. Electrospun nanofiber (ENF) deposition could be a potential method to improve osseointegration that can form extracellular matrix architecture on Ti.

Electrospinning is a process by which organized fibers of micro- to nanometer diameters can be deposited on a substrate from an electrostatically-driven jet of polymer solution through a needle [18,19]. Electrospun polycaprolactone (PCL) nanofibers with a high surface area-to-volume ratio are biocompatible and nontoxic, and can be used as a carrier for local drug delivery [19,20]. Khandaker and Snow developed an electrospin technique (U.S. Patent Application No. 14/734147, International Patent Application No. PCT/US15/45183, and U.S. Patent 9359694) by which a single strand of PCL NFM can be deposited on a titanium (Ti) implant [21]. Electrospun PCL NFM can be used to support the extracellular matrix for bone tissue generation inside the microgroove on a Ti surface. The application of the PCL electrospun fiber as an NFM coating material for Ti implants has not been investigated yet as a PCL NFM coating on the implant surface is poorly adhesive to the Ti surface. Khandaker and Riahinezhad recently developed (U.S. Patent Application No. 62312041) a set of steps by which PCL NFM can adequately be adhered to the microgrooves in Ti. The steps include plasma oxidation and a type I collagen (CG) coating on Ti. Collagen (CG) is a major insoluble fibrous protein in the natural extracellular matrix and in connective tissue [22]. Research shows that collagen-coated biodegradable polymer NFM has potential for both in vitro and in vivo cell growth [23,24]. 

The controlled fabrication of microgrooves on Ti surfaces and coating those microgrooves with CG-PCL NFM can improve the mechanical responses and biocompatibility properties of the implant. Coating microgrooves with CG-PCL NFM is associated with increased mechanical interlock, better cell adherence, higher bone-implant contact, and improved biomechanical interaction of Ti. The effect of coating the microgrooves with NFM on the mechanical stability and bone responses of Ti is not known. The objective of this study is to measure the effects of micro grooving and CG-PCL NFM treatments of Ti implants on the in vivo mechanical stability, bone density, and osseointegration of Ti.

## 2. Results

### 2.1. Nanofiber Characteristics

**Figure 1:**
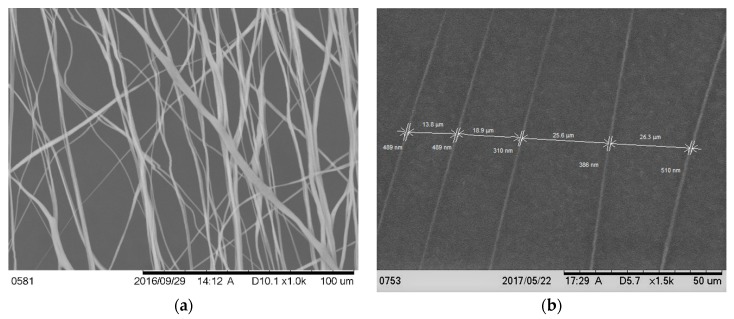
Scanning microscope image of the aligned poly-ε-caprolactone (PCL) nanofiber on a carbon type at (**a**) 1000× and (**b**) 1500× magnifications; (**b**) shows the diameter of the fiber and the distance between the fibers.

Figure 1a shows the scanning electron microscope images (magnification 800×) of the fiber on a carbon tape. The image shows that the fiber distribution was mainly unidirectional, although overlapping of the fibers along the direction was observed in several places. The diameter of the fiber on the carbon tape from the image (magnification 1500×) was found to be 438 ± 78 nanometers (Figure 1b). The distance between two fibers ranged from 13.8 to 26.3 μm. Because the fibers were collected on the carbon tape and Ti wires in a similar fashion, the diameter of the PCL fibers on the Ti wires were predicted to be in the micron to nanometer range. 

### 2.2. Ti Sample Characteristics

**Figure 2:**
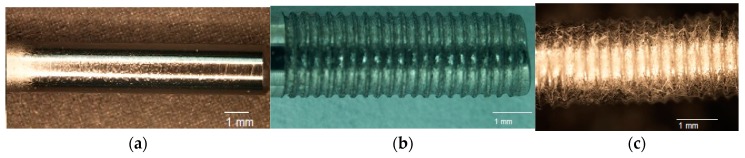
A fabricated (**a**) control; (**b**) groove, and (**c**) groove-NFM Ti samples.

**Figure 3:**
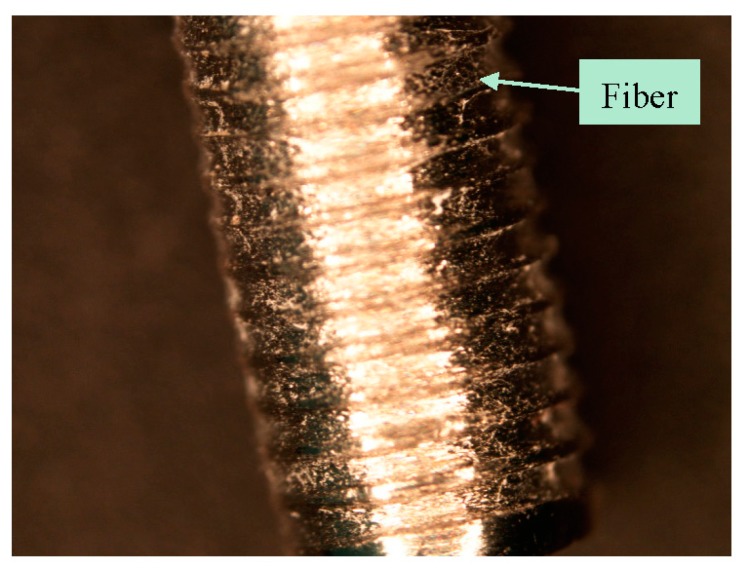
Attachment of collagen-poly-ε-caprolactone nanofiber mesh (CG-PCL NFM) coating after in the vivo implantation of the Ti wire after eight weeks.

The fabrication of controlled grooves by micromachining on a Ti surface and coating the grooves by multilayers of collagen PCL NFM was successfully conducted in this study. Figure 2a–c shows a fabricated sample from each group of test samples. A clear topographical difference among the samples was observed. The existence of the fibers at the groove was clearly visible in groove-NFM Ti samples (Figure 2c). Table 1 shows the geometric parameters of the fabricated Ti samples. The depth of the grooves varies from 71 μm to 88 μm, based on the measurements of three samples (Table 2). The width of the groove depends upon the Buehler Isomet wafer blade thickness, which was 150 μm. Since PCL fibers are biodegradable material, the post pull out observation of groove-NFM samples revealed the presence of a very negligible amount of PCL fibers at the microgrooves (Figure 3). The produced CG-PCL NFM was adequately attached with Ti for in vivo implantation. Since CG-PCL NFM resided inside the grooves, NFM would not have to withstand the shear load during the press fit of the implant into the rabbit leg. Therefore, the CG PCL NFM remained on the microgrooves after implantation. 

### 2.3. General Observations of Animals

All rabbits in this study remained in good health and did not show any recovery complications after surgery. There was an insignificant formation of callus and extraosseous ossification revealed on a few samples after cleaning the soft tissue from the bone. At retrieval, no visual signs of inflammatory or adverse tissue reactions such as swelling, redness, and tenderness in the surgery area were observed.

### 2.4. Mechanical Testing

**Figure 4:**
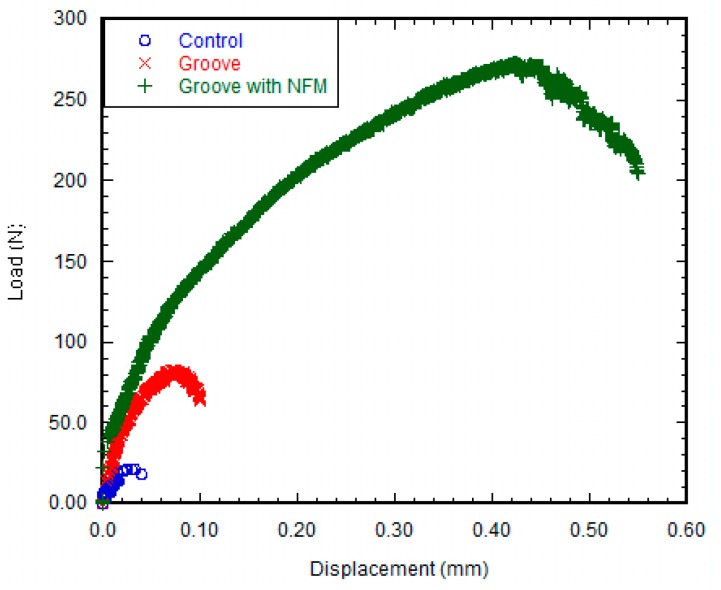
Load vs. displacement plot of a control, groove, and groove with NFM samples.

**Figure 5:**
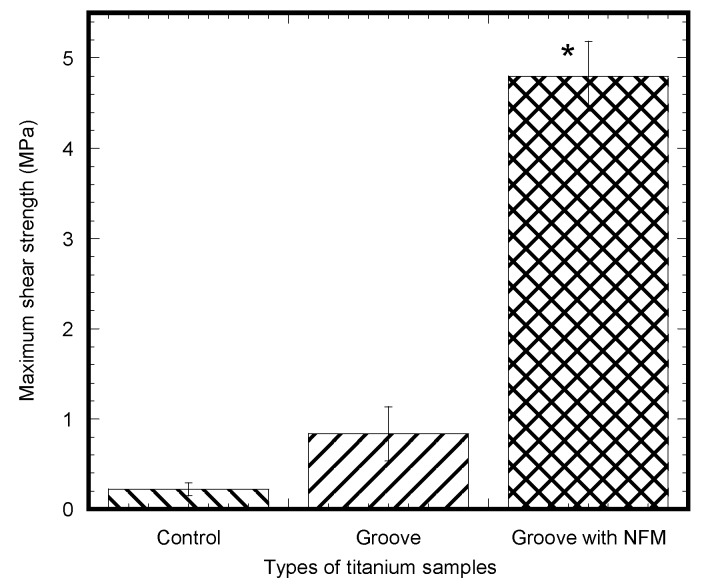
Pull out test results (in MPa) of different titanium samples after eight weeks of implantation. Bars represent mean ± SEM (*n* = 6). * represents statistical significant results compared to control samples for *p* < 0.05.

Figure 4 shows a clear difference in the load-displacement responses between the control and grooved samples. The load-displacement responses of the groove and groove-NFM Ti samples were characterized as elastic responses with a decrease of stiffness and a sudden failure of the specimen with a noticeable plastic response, whereas no noticeable plastic response was observed for the control Ti samples. This meant that deformation of the bone adjacent to the implant occurred for groove and groove-NFM Ti samples. Such deformation was found to be higher for grooves with NFM samples compared to other samples. There was no interfacial slipping (sudden fluctuation of the load without a noticeable displacement change) observed for any sample prior to failure during the mechanical testing. There was a steady decrease of the load with interfacial slipping, followed by a small increase of the load for both groove and groove-NFM samples after a failure of the sample. This was most likely due to bone adjacent to the grooves on Ti that was still in contact with the implant surface after a failure of the sample. On the other hand, a catastrophic decrease of the load was observed for control samples. Figure 4 also showed a clear difference of the maximum fracture load between NFM coated Ti samples and non-coated specimens. Figure 5 depicted the differences of maximum shear strength among the sample groups. Table 1 presents the differences of maximum shear strength with respect to the diameter, average groove depth, and the length of the implant in contact with bone among the Ti sample groups. The results show that the mean values of shear strength were three times higher for grooved Ti samples (0.84 ± 0.3 MPa, *n* = 6) compared to control samples (0.26 ± 0.09 MPa, *n* = 6), although the difference was not statistically significant (*p* > 0.05). When comparing the fracture strength results of the grooved Ti samples coated with CG-PCL NFM, we noticed that the ultimate shear strength of NFM coated grooved samples (4.79 ± 0.39 MPa, *n* = 6) was higher (more than 18 times) compared to the control samples (*p* < 0.05). No statistically significant differences were observed among the sample group for the diameter (*p* > 0.05). Additionally, there were no statistically significant differences when considering the length of the implant in contact with bone among the sample group (*p* > 0.05). Therefore, the surface coating of the Ti samples by CG-PCL NFM had a significant effect on the shear strength of the samples. Post-test visual inspection of the specimens revealed that failure typically occurred at the bone-implant interfaces.

### 2.5. Histomorphometric Analysis

**Figure 6:**
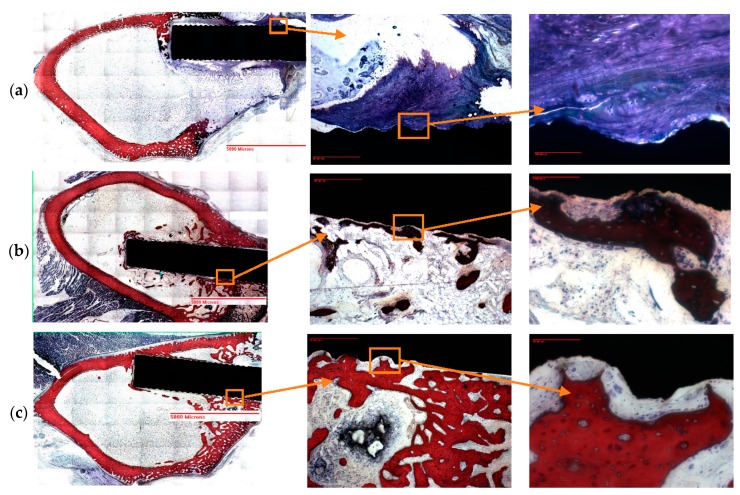
Stained images of decalcified Ti-bone samples: (**a**) groove and (**b**,**c**) groove-NFM. In the image, older cortical bone has a lighter pink color and newer cortical bone stains dark red, trabecular bone is stained in blue, while connective tissue is stained in white. In the above figures, the first, second, and third column images were taken at 0.5×, 4× and 20× magnifications. New cortical bone growth along the microgroove is visible from both groove-NFM samples, whereas connective tissues and trabecular bone growth along the microgroove is visible from groove sample.

The groove and groove-NFM Ti samples were intact in the bone during the histology process. The histomorphometric analysis results (Figure 6 and Table 2) showed that the amount of bone and tissue growth on the groove-NFM Ti samples was higher compared to the groove Ti samples. All histomorphometric parameters were increased for the groove-NFM Ti samples compared to the groove samples. Bone ingrowth to the microgroove for each sample was clearly visible in the stained images (Figure 6a–c). Although new bone formation was observed in all bone samples, there is a distinct difference in the type of new bone formation between the groove-only and groove-NFM Ti samples. The groove-only sample had mostly new trabecular bone tissue (Figure 6a), whereas both new cortical and trabecular bone tissue were observed along the implant surface for both groove-NFM samples. Most importantly, the migration of cortical bone tissue was observed in the microgroove for both groove-NFM Ti samples (Figure 6b,c), which may be the main cause for the increased mechanical stability of groove-NFM Ti samples in comparison to the groove Ti sample.

### 2.6. μCT Analysis

**Figure 8:**
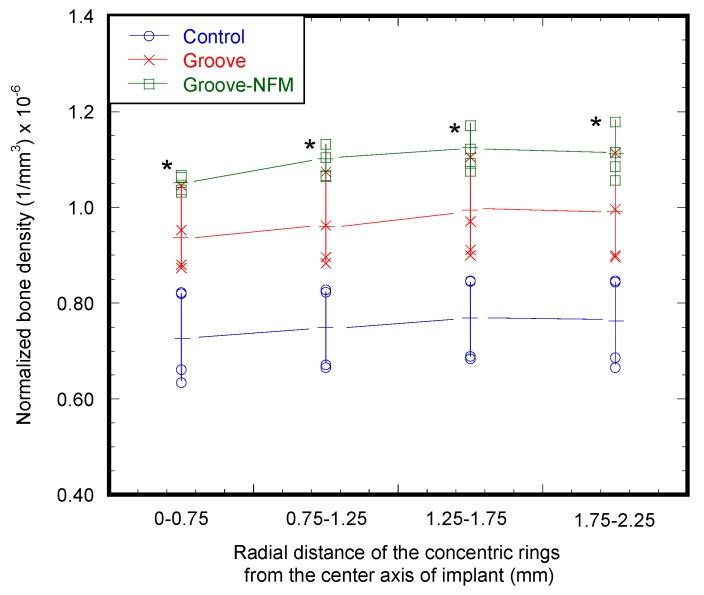
Normalized bone density (total bone density/weight of the rabbit) belonging to the volume of the concentric cylindrical rings from the center axis of the implant. * represents statistically significant results compared to control samples for *p* < 0.05.

The calibration equation using a bone densitometry phantom block was determined as *y* = 0.0002*x* + 0.9091, where *x* is the mean value of voxels within the region of interest (ROI) and *y* is the bone density in g/cm^3^. This equation was used for the calculation of the bone density of all test samples. Figure 7 shows the micro-CT images of bone samples for the determination of the bone density adjacent to the implant. Table 3 shows the trabecular and cortical bone density in g/cm^3^ of a rabbit femur prior to implantation. Table 3 also presents the trabecular and cortical bone density of the control (weight = 3.73 ± 0.37 kg, *n* = 3), groove (weight = 2.82 ± 0.25 kg, *n* = 3), and groove-NFM (weight = 2.46 ± 0.09 kg, *n* = 3) samples adjacent to the implant holes. The ranges of trabecular and cortical bone density of each group of test samples overlaps with the values of bone density of the rabbit without the implant. Therefore, our method of implantation did not have any negative influence on the bone remodeling process. Figure 8 shows the variation of normalized bone density (total bone density/weight of the rabbit) belonging to the volume of the concentric cylindrical rings from the center of the implant. The normalized bone density around the implant containing grooves was significantly higher than the control samples (*p* < 0.05). By coating the grooves with CG-PCL NFM, a further increase in the normalized bone density was observed. The normalized bone density around the groove implant containing CG-PCL NFM was higher than the groove samples, but the difference was not statistically significant (*p* > 0.05). The higher bone density of groove-NFM around the implant may be the reason for the higher mechanical stability for groove-NFM samples compared to the other group of samples.

## 3. Discussion 

Implant failure due to poor fixation of the implant with the biomaterial is a common problem in various orthopedic and orthodontic surgeries. Implant fixation mostly depends upon the implant surface topography. In the present study, the mechanical stability of a groove and CG-PCL NFM coated implant with joining biomaterial was found to be significantly higher compared to the control and only groove samples from the mechanical tests. The histological analysis revealed a clear positive effect on the implant-bone contact geometry and amount of new bone formation due to the coating with CG-PCL NFM. 

The pull out tests showed that the implant stability, quantified by the maximum shear strength, significantly improved due to the coating (Figure 5). Our results are supported by several lines of evidence: (1) controlled grooves significantly increase the mechanical interlock between the implant and joining biomaterial [5]. In addition, cells are especially responsive to groove architecture (particularly shape and direction) and on such surfaces cells aligned and migrated along the groove direction [25,26]; (2) Microscale surface features on implants, such as microroughness [27] and microporosity [28], can act as potent modulators of cellular function through the onset of focal adhesion formation. Microscale roughness of the titanium implant surface was created from the roughening of the surface by micro machining; (3) Grooves with collagen coated PCL NFM protein, in combination with controlled roughness, can further influence the adhesion, proliferation, and differentiation of cells on Ti. 

Post pull out observations of the groove-NFM samples revealed the in vivo degradation of PCL nanofibers. A very negligible amount of fibers remained at the Ti/bone interface (Figure 3). It is important to mention that the observation was made after eight weeks of in vivo implantation of the Ti sample and the sample was cleaned with water before imaging. Therefore, the CG-PCL NFM biocoating technique developed in the study created adequate adhesion with Ti at the groove and degradation.

The mechanical responses (load vs. displacement) of the groove only and groove-NFM Ti samples exhibit comparable mechanical responses of natural tissue-tissue joints such as bone-cartilage [29] and dentin–enamel [30], where there is a significant reduction in the stress level sustained beyond the maximum yield strength point. Decreasing post-yield loads with increasing displacement that occurs during “post yield softening” was observed for both groove and groove-NFM Ti samples, as shown in Figure 4. The increase in the deformation of the bone adjacent to the implant with the increase of the load could be the reason for the decrease of interfacial stiffness for groove and groove-NFM Ti samples. The exact fracture mechanism of groove and groove-NFM Ti/bone samples has not been investigated. In contrast, control samples, having low interdigitation sites due to smooth polishing, exhibited no post-yield softening behavior under the pull out tension load. Therefore, lower forces were required to cause failure of the control Ti/bone interfaces than groove Ti/bone interfaces. 

The present study evaluated osseointegration by an analysis of the implant-nondecalcified bone contact surface by a histomorphometric analysis of the percentage of bone and connective tissue formation with the implant surface. The histomorphometric analyses confirmed bone in-growth into the microgroove. In addition, analysis showed that the amount of new bone generation, particularly cortical bone, on the Ti implant increased significantly due to the CG-PCL NFM coating. μCT analysis results show a clear positive effect on the bone density for the implant coated with CG-PCL NFM. The bone density values around the implant for all test samples were in the range of 1.21 to 1.60 g/cm^3^ (Table 3), which are in the range of rabbits without an implant and healthy bone density for rodents (higher than > 0.4 g/cm^3^ [31,32]).

The enhancement of the bone formation around the CG-PCL NFM coated Ti implant was due to the fact that the fiber coating improved the surface and cytocompatibility properties of Ti. In our previous study, we have determined the in vitro effects of CG-PCL NFM coated Ti samples on the surface topography and cytocompatibility (osteoblast cell adhesion, proliferation, mineralization, and protein adsorption) properties. Our study found that the CG-PCL NFM coating on Ti improved the surface roughness, osteoinductive (osteoblast cell adhesion, proliferation, mineralization, and protein adsorption) properties of Ti. Our study agrees with other studies where it has been reported that when implants are coated with a functional fiber coating, it increases the osteoinductive properties of the implant, thereby improving osseointegration of the implant [33].

To our knowledge, this is the first study to evaluate the effect of CG-PCL NFM coating treatment on the mechanical stability and osseointegration of a titanium implant using a rabbit model. This study used the maximum shear strength as the criterion to compare the mechanical stability of different sample groups. Several reports have used maximum pull out forces for evaluating the mechanical stability of implant/bone samples [34,35]. Since the mechanical stability, quantified by shear strength, depends upon the contact surface area between the Ti and bone, the pull out force is not an appropriate parameter to compare the effect of a treatment method on the mechanical stability of Ti. Boone et al. [36] found that the maximum shear strength of hydroxyapatite (HA) coated thermoplastic implants after four and 12 weeks was 5.9 and 8.2 MPa, respectively. In our study, mechanical tests on the CG-PCL NFM coated Ti implant showed that the maximum shear strength of NFM coated Ti/bone samples eight weeks post-surgery was 4.79 ± 0.39 MPa. This difference of shear strength is likely due to the dissimilarity of the implant material. In our study, Ti was used as the implant material, whereas thermoplastic implants were used as the implant by the previous authors. Zankovych et al. [37] found that the maximum shear strength of the Ti/bone interface in a rat model after eight weeks of surgery was 0.15 to 0.6 MPa. These results are in agreement with the maximum shear strength of the Ti/bone interface for the control Ti samples (0.26 ± 0.09 MPa) found in our study. 

Our study found that the mean value of shear strength of the groove Ti samples was three times higher in comparison to control Ti samples, although the difference was not statistically significant due to the large deviation of the test data. An and Draughn [8] reported various factors that affect the in vivo mechanical strength of the implant/bone interfaces. In our study, surgery (e.g., diameter and depth of the hole created in bone by hand drill), mechanical test factors (e.g., dissection of soft tissue from the bone, pre-load applied to the Ti/bone samples during the curing of cement), and biological factors (e.g., porosity, density, and mineralization of bone) are some of the factors that might cause large deviations within mechanical test data. 

This study is limited to static pull out tension tests on different groups of Ti/bone samples. The strain rate used for these studies was 0.3 mm/min or 0.005 mm/s. This strain rate was selected due to the fact that violent fractures occur at strain rates of between 0.3 mm/min and 1 mm/min [32]. Since large increments of load occur at the initial stage of the mechanical tests, the study can collect more data at this stage using the 0.3 mm/min strain rate. This is important for determining the mechanical responses of the implant-bone joint, particularly for control samples, where small forces were required for the breakage of the samples. No histomorphometric analyses was conducted on control samples, since the mechanical test results clearly show a very poor integration of control Ti with bone in comparison to groove and groove-NFM samples. Therefore, randomly selected samples from the groove and groove-NFM groups were tested at an external facility (University of Alabama at Birmingham) by an expert histology technician. Since the goal of this study was to establish a basis for further exploration of the microgroove-assisted NFM coating technique, a small sample size was used to determine the type and amount of bone and connective tissue in contact with the implant surface. Further investigation of the effect of the CG-PCL NFM coating on in vivo differentiation and gene expression is required to understand the osteogenesis pathways and activities induced by NFM. Such studies can greatly facilitate the understanding of bone repair by the NFM. Since the purpose of the histomorphometric analysis was to evaluate CG-PCL NFM effect on the osseointegration of Ti, a histomorphometric analysis was conducted on a larger number of groove-NFM samples compared to groove samples. The design of the proposed surface modification treatment using Ti implants is limited to its applications for healthy bone. However, since local delivery of the drug with an NFM coating on the surface modification technique is possible, this technique may also be beneficial to diseased (osteoporotic) tissue. 

This study is important for the development of a novel surface modification technique for orthopedic implants. A potential opportunity for the improvement of in vivo tissue to implant osseointegration and faster healing times is possible through the combined tailoring of interdigitation sites through grooving, electrospun nanofiber-based extracellular matrix protein layers, and microgrooving incorporated surface roughness to the implant. Such a technique can also be applied for the biological fixation or functional integration of other orthopedic implant materials. In addition, the results of the study are useful for implant design by providing an understanding of how failure responses of the Ti/bone interfaces can be improved by extra cellular matrix adhesion and surface modification of the implant materials.

The novelty of this study is the evaluation of microgroove and CG-PCL NFM treatment on a titanium alloy for improving its mechanical and biological responses. Specifically, this study measured the effect of treatments on the in vivo biocompatibility of a Ti alloy (Ti-6Al-4V Eli) and the mechanical strength of the Ti alloy with bone. This article contributes to the applied biomaterials community by providing an increased understanding of how the failure functions of the titanium implant-bone interfaces can be improved by a novel biocoating approach. Microgrooves that are produced from machine sawing on Ti are uncontrolled and this technique might not be suitable for Ti implants with a complex shape. A greater and more controlled Ti–bone contact area can be created by plasma ion deposition or etching at selected regions on Ti. Plasma ion deposition or etching techniques can also be applied to Ti implants with a complex shape. This work could be extended by a study of the effect of microgrooves on the biomechanical functions of Ti, as created by the previously mentioned process and coated with NFM.

## 4. Materials and Methods

### 4.1. Materials

Ti wires (6Al-4V ELI, ASTM B 348 standard, grade 23, biocompatible) of a 2.26 mm diameter and 12 mm length were purchased from Supra Alloys Inc., Camarillo, CA, USA. PCL solution was prepared by mixing PCL pellets (pellet size ~3 mm, average Mn 80,000) with acetone (laboratory reagent ≥99.5%). Both PCL and acetone were purchased from Sigma Aldrich (Sigma-Aldrich Co. LLC., St. Louis, MO, USA). Rat tail type I collagen (Discovery Labware Inc., Bedford, MA, USA) was purchased from Dow Corning (Midland, MI, USA).

### 4.2. Specimen Design

**Figure 9:**
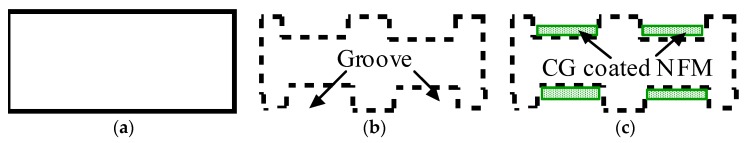
Schematic representation of the longitudinal section images of (**a**) control, (**b**) groove, and (**c**) groove-NFM samples.

Three groups of implants were used in this study: control, groove, and groove-NFM, as schematically represented in Figure 8. Ti implants without grooves and NFM were named control samples (Figure 9a). Ti samples with machined microgrooves along the circumferential surface were named groove samples (Figure 9b). Ti samples with microgrooves with CG-PCL NFM were named groove-NFM samples (Figure 9c). 

### 4.3. Preparation of Ti Samples 

#### 4.3.1. Control Samples

All groups of Ti samples were polished using a drill machine chuck and gripper. A Ti wire was secured at the drill chuck. A polish paper (10 mm × 50 mm) was wrapped around the Ti wire with pressure using the gripper of the drill machine. Polishing occurred when the drill machine was in operation. All groups of Ti wires were polished in order to have a uniform surface morphology for all test samples. Ti wires samples were circumferentially polished up to 8 mm from one end. The three steps polishing technique, as recommended by Buehler [38], was used to polish the Ti surface. The first step was to polish the Ti surface using CarbiMet 2 Abrasive paper (Buehler, Lake Bluff, IL, USA) with 9 μm MetaDi supreme diamond suspension (Buehler, Lake Bluff, IL, USA) spray. The second step was to polish the Ti surface again using a Buehler Ultra Pad cloth with 0.05 μm MetaDi Supreme Diamond Suspension spray. The third step was to polish the Ti surface using Buehler MicroCloth with MasterPrep Alumina polishing suspension spray. Each polishing step was conducted for 2 min. Ti wires were cleaned by ethanol after each step and finally autoclaved at 120 °C for an hour. Ti wires without further treatment were considered as control samples.

#### 4.3.2. Groove Samples

A diamond saw blade (Isomet wafer blade 76.20 mm diameter × 0.15 mm thickness, 15HC (Buehler, Lake Bluff, IL, USA)) was used to machine the microgroove on the circumferential surface of the wire. The Ti wire was fastened with the shaft of a motor. The motor was secured in the saw machine at the sample grip holder. Each microgroove was created by running the motor and saw machine simultaneously in opposite directions for 8 s. Eighteen bands of circumferential parallel grooves were created, starting at a 0.5 mm distance from one end of the Ti wire. The microgrooves were 0.05 mm apart from each other. All samples were cleaned in an ultrasonic cleaner followed by 70% ethanol and then autoclaved at 120 °C. Ti wires with only microgrooves were classified as groove samples.

#### 4.3.3. Groove-NFM Samples

**Figure 10:**
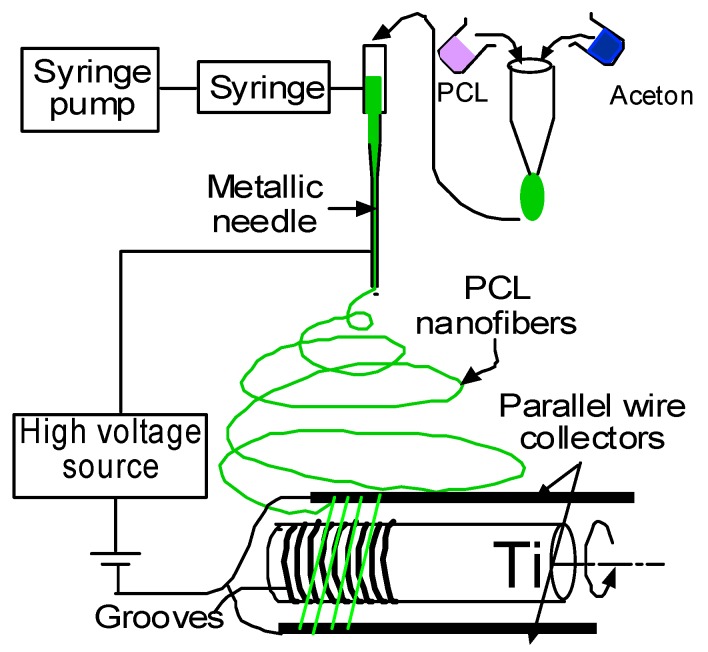
Schematic representation of coating groove Ti samples by aligned electrospun nanofiber using an electrospin process.

The autoclaved Ti samples with grooves were exposed to plasma O_2_ for 5 min at Frequency 40 kHz and power 30 watt in a Zepto low pressure reactive ion etching system (Diener electronic GmbH + Co. KG, Ebhausen, Germany) to increase the attachment of collagen to the Ti surface [23]. Ti wire was soaked with a collagen solution and PCL electrospun nanofiber was deposited on the Ti. Aligned PCL nanofibers were deposited on the grooved Ti samples using an electrospin setup (Figure 10). The details of the fabrication of the PCL electrospun nanofiber can be found in Khandaker et al. [39]. In short, PCL pellets (7.69 wt %) were mixed with acetone in an ultrasonic (Sonics & Materials, Inc., model # Vibra-cell VCX 130, Newtown, CT, USA) mixer. The sonication process was carried out at approximately 60 °C for 30 min. The solution was poured into a glass syringe on an infusion pump (Harvard Apparatus, model # PHD ULTRA, Holliston, MA, USA) for PCL fiber production. PCL melt was ejected from the glass syringe through an electrically charged needle (23G blunt needle, 1 in length, model # BX 25). The needle was charged by a high voltage power source (Gamma High Voltage Research, Inc., model # ES 30 series, Ormond Beach, FL, USA). Aligned PCL fibers were collected between two parallel wires. Since the color of PCL fibers is white and Ti is a non-conductive material, it is not possible to analyze the topography of fibers on Ti using a scanning electron microscope (SEM). It creates a bright white image due to the reflection of light on fiber coated Ti samples during SEM imaging. Therefore, the topography of fibers was analyzed by wrapping the Ti by carbon tape using TM3000 TableTop Scanning Electron Microscope (Hitachi High Technologies America, Inc., Schaumburg, IL, USA). The fibers were collected on the tape by manually rotating the Ti sample, just like the fibers were collected on Ti during the preparation of in vivo test samples. 

Collagen solution was prepared by mixing 2.3 microliters of type I collagen with 0.23 microliters of acetic acid (0.02 M) and 195 microliters of deionized water in a vortex mixer. Ti wire was soaked with the collagen solution. Aligned PCL ENF were deposited on the Ti by manually rotating the Ti wire six times and were dried in a UV chamber. Finally, the CG solution coating on the Ti was applied again and dried to prepare the groove-NFM Ti samples. The groove-NFM samples were kept at 4 °C until implantation in the rabbit femur.

### 4.4. Animal Study

#### 4.4.1. Sample Design

Each group of Ti samples was implanted immediately proximal to the lateral femoral condyle of six to eight week-old either New Zealand White Rabbits (NWR) at the University of Oklahoma Health Science Center (OUHSC) animal care facilities, according to the approved IACUC protocol (14-055-SSX). A total of 18 NWR were used for this study. Each rabbit received two samples of the same group of Ti, with one Ti sample per leg. Six samples from random rabbit legs were used for pull out tension tests per group to produce a statistically independent sample for each sample group. This sample number was determined from the power analysis of Peter et al. [32] in vivo mechanical stability test data to present the statistical significant outcome. The other implant on each rabbit was reserved for histology or separate studies. Two different animal legs were CT scanned before surgery for the determination of CT threshold values and bone density without the implant. Four samples were randomly selected from each group of tension test samples after surgery for the determination of the bone density of the test samples. 

#### 4.4.2. Surgery Protocol

Rabbits were anesthetized with ketamine and xylazine by intramuscular injection and transported to the surgical preparation room. Rabbits were given buprenorphine and oxytetracycline, subcutaneously, and were tattooed on an inner ear. The entire circumference of both rear legs proximal and distal to the distal femur of the rabbits was shaved, and the leg was then prepped with chlorhexidine scrub, alternating with alcohol. The rabbits were then moved to the operating room. After a lidocaine drip to the back of the mouth, rabbits were intubated with a 3 mm ID endotracheal (ET) tube. Isoflurane was administered with oxygen via the ET tube. A final surgical prep was then performed. A total of 0.5 mL of 0.5% lidocaine was injected subcutaneously at the incision site. Surgery started once the rabbit was at a completely surgical plane of anesthesia.

**Figure 11:**
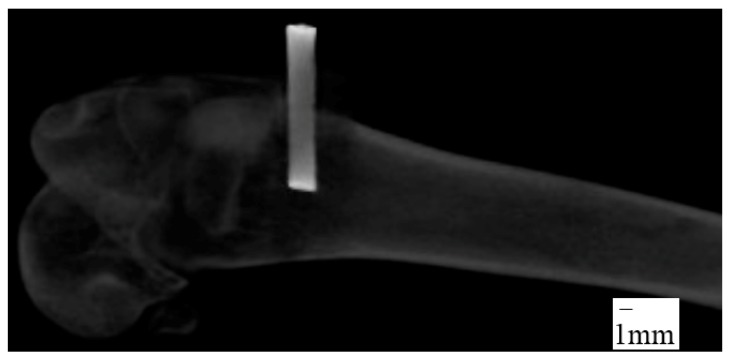
X-ray image of femur after surgery.

A 2.5 cm curvilinear midlateral skin incision was made, starting at the distal end of the body of the femur and extending distally to just below the level of the patella. The fascia lata was then excised in line with the skin incision. The vastus lateralis muscle was retracted cranio-medially to expose the lateral condyle and the joint capsule opened sharply. A 1.96 mm diameter drill bit was used to drill a 6~8 mm depth hole perpendicularly into the lateral condyle by a manual hand drill press and then Ti wire sample was hand-pushed into the hole. The joint capsule, muscle layers, and subcutaneous tissue were then sutured with 4-0 vicryl in a continuous pattern and the skin was closed with a subcuticular pattern. X-ray images of the implantation sites were taken immediately after surgery to measure the depth of implant inside the bone (Figure 11). All rabbits received buprenorphine post-operatively for three days and were closely monitored for food and water intake, fecal output, and healing properties. Rabbits were euthanized eight weeks after surgery. Soft tissues were carefully dissected off the bone. The Buehler saw machine was used to trim the Ti-bone samples to an adequate size for mechanical and histological experiments. The samples were kept moist during the preparation by soaking with saline.

### 4.5. Pull out Tension Tests

The Ti-bone sample was embedded in a custom made cylindrical cup using a low-viscosity acrylic bone cement in the Test Resource universal testing machine (precision of load cell = ±0.0003 N). The acrylic cup was used to permit the coaxial alignment of the implant in the direction of the pull-out force. The bottom of the cup was fastened in the bottom of the test machine. The implant was carefully fastened to the top gripper in the mechanical tester and slowly lowered to embed the Ti-bone sample in a low-viscosity acrylic bone cement (BioMedtrix veterinarian bone cement) and was cured for an hour. Pull out tension tests were conducted on each sample at room temperature with a steady speed of 0.3 mm/min [32] until the breakage of the implant from the bone. The maximum pull-out force from the load-displacement curve was measured and the mechanical stability (quantified by maximum shear strength) was calculated from the ratio of the force at the point of breakage of the implant from the bone and the surface area of Ti in contact with bone. The depth of the grooves in grooved samples were approximately 70 micrometers, which is much lower than the diameter of the sample. Therefore, the surface area of both grooved and non-grooved Ti samples was measured by π × *D* × *L*, where *D* is the diameter of the samples and *L* is the length of the implant in contact with the bone.

### 4.6. Histomorphometry Analyses

Groove and groove-NFM Ti samples were selected randomly for the histomorphometry analyses. Sectioning, staining, and imaging for histomorphometric analysis was done at a pathology core research laboratory at the University of Alabama at Birmingham (UAB). Because the samples were frozen, the trimmed samples were fixed in 70% ethanol solution by soaking the samples in the solution in a shaker (VWR orbital shaker) at the rate of 70 Hz for 48 h before shipping to UAB for the histology and histomorphometry analyses. Thin histologic sections were made of nondecalcified bone with titanium implants. Histological processing consisted of increasing concentrations of ethanol followed by several changes of xylene. The tissue was then infiltrated with four changes of a 95% Methyl Methacrylate (MMA) and 5% Dibutyl phthalate (DBP) solution. The sample remained in each solution for one week. After infiltration, the samples were embedded in a solution of 95% MMA and 5% DBP, with 0.25% perkodox used as a catalyst for polymerization. Once polymerized, the blocks were exposed to UV light overnight for final curing. The fully polymerized (plasticized) sample blocks were cut out of their embedding molds and shaped for the proper orientation by rough cutting with a band saw and the fine adjustments were made using a tabletop grinder. Once the desired cutting plane was parallel to a flat surface, the sample was glued onto a backing slide using a Technovit 7210 VLC (Kulzer GmbH, Wehrheim, Germany). The backing slide and sample block were then mounted and cut, approximately 500 microns away from the edge of the implant, using the Exakt diamond blade apparatus. The block was then placed on the vacuum head of the Exakt table grinder and ground down to the desired plane. A final slide was glued on the cut and ground surface, once again using the Technovit 7210 VLC. Prior to attaching the final slide. The block and backing slide thickness was measured and recorded as value “A”. The final slide thickness was recorded as Value “B” and once the backing slide, block, and final slide were all glued together, the “sandwich” thickness was recorded as Value “C”. The glue thickness “G” was calculated using the following equation: G = C − (A + B). The “sandwich” was then remounted onto the diamond-coated band saw system and approximately 500 microns was cut from the final slide. The thickness of the sample was calculated on the final slide “X” from the known thickness of the glue and final slide by measuring the thickness of the final slide that was cut from the backing slide sandwich “D”: X = D − (B + G). The final slide was placed back on the Exakt table grinder and ground to a final thickness of approximately 50 microns using incrementally finer sandpaper. The final slide was polished and then stained with Sanderson’s Rapid Bone Stain (SRBS).

All slides were analyzed using the imaging analysis software Bioquant Osteo 2015 Version 15.1.60 (Bioquant^®^ Image analysis corporation, Nashville, TN, USA). A template was designed to measure the total area of tissue 100 microns off the surface of the implant and within the healing callus of the cortical bone. All of the bone area and perimeter within that region was measured and recorded as either trabecular bone or cortical bone. The total length of the implant was also measured as any surface that was in contact with bone. The average depth of the implant grooves was also measured. A number of histomorphometric parameters were measured: the groove depth, percentage of bone to implant contact, total new bone surface area, total cortical bone surface area, and percentage of bone volume over total volume (BV/TV).

### 4.7. μ-CT

**Figure 12:**
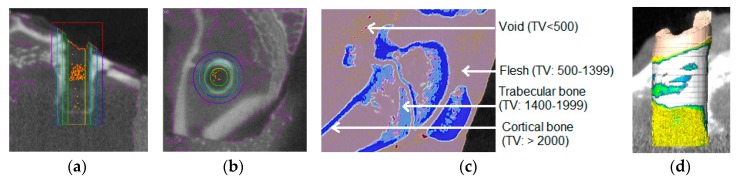
Process to find the bone density from a selected region of interest (ROI) of a μ-CT image: (**a**) column selection around the center of the implant hole. Bottom of column is 500 μm from the bottom of the implant hole. The columns (represented by different color) were created by a transverse segmentation of the bone section into three rings. The inner section represents the holes created by an implant; (**b**) concentric ring creation around the hole of the implant. The three rings with radii of 2.25–1.75, 1.75–1.25, and 1.25–0.75 mm were represented by colored circles; (**c**) determination of CT threshold values to identify different types of bone tissue from a CT scan image of a rabbit femur bone before surgery (without implant). The CT scanning was conducted around the same location where the implant was inserted. (**d**) Use of density thresholds to assign portions of column to the specific bone tissue to generate 3D volume of the specified ROI.

Nine Ti samples were scanned by μ-CT after the pull out tension tests to examine the bone density around the implant site. The calibration equation for the calculation of the bone density of test samples was derived by μ-CT scanning a bone densitometry phantom block (QRM-MircoCT-HA Phantom (QRM GmbH, Moehrendorf, Germany)). A Gamma Medica X-PET XO machine (Gamma Medica, Salem, NH, USA) was used at 80 KeV with slices every 50 μm for μ-CT scanning. Using the AMIRA 5.6 software, a threshold of 1400+ was selected to delineate the bone and the edges were smoothened for the 3D volume using a size of three. A column with a radius of 2.25 mm centered with an implant hole was selected (Figure 12a). This column was segmented into three rings with radii of 2.25–1.75, 1.75–1.25, and 1.25–0.75 mm and a single column with a radius of 0.75 mm (Figure 12b). These were each further segmented into four sub-regions using thresholds: Cortical bone: 2000+, Trabecular bone 1400–1999, Flesh: 500–1399, and Empty space: <500. These values were determined by scanning a rabbit femur at the distal end of the body of the femur of a rabbit (weight 2.25 kg) prior to implantation (Figure 12c,d). Since bone density depends upon the age of the animal, normalized bone density was calculated by dividing the total bone (cortical and trabecular) amount over the weight of the animal before necropsy.

### 4.8. Statistical Analysis

Experimental results were reported in this study as mean ± standard error of mean (number of samples) for each sample group. A one-factor analysis of variance (ANOVA) with subsampling assuming unequal variances was performed using the statistical tools of KaleidaGraph 4.02 version software (Synergy Software, Reading, PA, USA) to determine if there was any significant effect of the application of microgrooves and NFM on the mechanical stability and bone density of Ti. For all statistical tests, *p* < 0.05 was considered as statistically significant.

## 5. Conclusions

This study established a proof of concept of microgrooving on Ti and a CG-PCL coating of the grooves for improving the mechanical stability and osseointegration of an orthopedic implant. This study shows that the combined application of microgrooves and an extracellular matrix structure enhanced the bone density, mechanical interlock, and osseointegration of an implant with the host tissue. The fabrication of surface microgrooves on Ti and local delivery of extracellular matrix protein (collagen) at the microgrooves sites allowed the increase of the mechanical interlock and implant–bone contact area of a Ti implant. The histologic examination confirmed that our novel technique of a CG-PCL NFM coating on Ti can improve the osseointegration of Ti under load-bearing conditions in vivo. Moreover, the present study showed that the peri-implant new bone volume is increased due to the CG-PCL NFM coating. Therefore, implant surface modification by a groove-associated bio coating can potentially improve the mechanical and biological responses of orthopedic implants with surrounding biomaterials. Orthopedic implants that can deliver vitamins, proteins, and minerals may then represent an interesting approach for healthy and osteoporotic patients in need of a total joint replacement.

## Figures and Tables

**Figure 7:** Micro-CT images of bone samples for the determination of the bone density adjacent to the implant. In the images, the length of bar is 2.2 mm.

**Table:** 

Types of Sample	Sample Number			
	1	2	3	4
Control	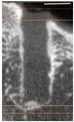	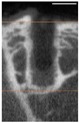	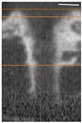	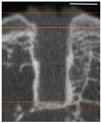
Groove	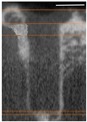	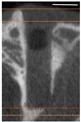	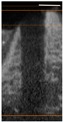	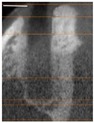
Groove-NFM	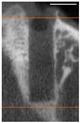	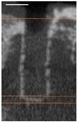	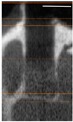	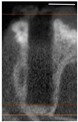

**Table 1:** Geometric parameters of Ti samples and maximum shear strength for different groups of Ti-bone samples. The values are given as means ± SEM (*n* = 6 each). * Significantly greater than control (*p* < 0.05); ^†^ Significantly greater than groove.

Sample Type	Diameter of Implant (mm)	Length of Implant in Contact with Bone (mm)	Surface Area (mm^2^)	Maximum Shear Strength between Titanium and Bone (MPa)
Control	2.20 ± 0.00	6.63 ± 0.32	45.88 ± 2.21	0.26 ± 0.09
Groove	2.17 ± 0.02	5.68 ± 0.20	38.70 ± 1.59	0.84 ± 0.3 *
Groove with NFM	2.19 ± 0.01	5.57 ± 0.1	38.28 ± 0.65	4.79 ± 0.39 *^,†^

**Table 2:** Histomorphometric analysis data for a randomly selected groove Ti sample and two groove-NFM Ti samples.

Histological Analysis Parameters	Groove	Groove-NFM	
		Sample 1	Sample 2
Weight of animal before necropsy (kg)	2.55	2.62	2.65
Groove depth (μm) *	75.44 ± 0.28	88.10 ± 0.34	71.44 ± 0.11
% Bone to Implant Contact (%)	39.78	62.18	90.60
Total New Bone Area (mm^2^)	0.02	0.36	0.29
Total Cortical Bone Area (mm^2^)	0.00	0.15	0.26
Cortical Bone Surface (mm)	0.00	3.59	7.81
New Bone Surface (mm)	1.29	11.23	13.12
Total Tissue Area in the ROI (mm^2^)	1.84	1.94	2.17
Total BV/TV (%)	1.01	26.18	25.63
Total New Bone/TV (%)	1.01	18.52	13.54
Total Cortical Bone/TV (%)	0.00	7.66	12.09

* There are 18 grooves per sample. Depth of all grooves were reported as mean ± SEM.

**Table 3:** Trabecular (Threshold value: 1400–1999) and cortical (Threshold value: >2000) bone density values in g/cm^3^ for different test samples as a function of the cylindrical rings region around the implant. The values for control, groove, and groove-NFM samples are given as means ± SEM.

Cylindrical Rings Region (mm)	Tissue Types	Sample Types			
		No Implant (*n* = 2)	Control (*n* = 4)	Groove (*n* = 4)	Groove-NFM (*n* = 4)
0–0.75	Trabecular	1.245 ± 0.005	1.218 ± 0.007	1.225 ± 0.005	1.233 ± 0.006
	Cortical	1.394 ± 0.013	1.410 ± 0.030	1.405 ± 0.023	1.394 ± 0.037
0.75–1.25	Trabecular	1.242 ± 0.009	1.235 ± 0.003	1.240 ± 0.002	1.241 ± 0.001
	Cortical	1.397 ± 0.015	1.437 ± 0.013	1.411 ± 0.016	1.475 ± 0.025
1.2–1.75	Trabecular	1.243 ± 0.011	1.238 ± 0.004	1.246 ± 0.000	1.239 ± 0.001
	Cortical	1.399 ± 0.009	1.472 ± 0.025	1.441 ± 0.031	1.503 ± 0.036
1.7–2.25	Trabecular	1.242 ± 0.015	1.231 ± 0.007	1.245 ± 0.002	1.232 ± 0.005
	Cortical	1.393 ± 0.012	1.454 ± 0.022	1.429 ± 0.034	1.479 ± 0.050
